# Open source software for automatic detection of cone photoreceptors in adaptive optics ophthalmoscopy using convolutional neural networks

**DOI:** 10.1038/s41598-017-07103-0

**Published:** 2017-07-26

**Authors:** David Cunefare, Leyuan Fang, Robert F. Cooper, Alfredo Dubra, Joseph Carroll, Sina Farsiu

**Affiliations:** 1 Department of Biomedical Engineering, Duke University, Durham, NC 27708 USA; 2 Department of Ophthalmology, Scheie Eye Institute, University of Pennsylvania, Philadelphia, PA 19104 USA; 3 Department of Psychology, University of Pennsylvania, Philadelphia, PA 19104 USA; 4 Department of Ophthalmology, Stanford University, Palo Alto, CA 94303 USA; 5 Department of Biomedical Engineering, Marquette University, Milwaukee, WI 53233 USA; 6 Department of Ophthalmology & Visual Sciences, Medical College of Wisconsin, Milwaukee, WI 53226 USA; 7 Department of Ophthalmology, Duke University Medical Center, Durham, NC 27710 USA

## Abstract

Imaging with an adaptive optics scanning light ophthalmoscope (AOSLO) enables direct visualization of the cone photoreceptor mosaic in the living human retina. Quantitative analysis of AOSLO images typically requires manual grading, which is time consuming, and subjective; thus, automated algorithms are highly desirable. Previously developed automated methods are often reliant on ad hoc rules that may not be transferable between different imaging modalities or retinal locations. In this work, we present a convolutional neural network (CNN) based method for cone detection that learns features of interest directly from training data. This cone-identifying algorithm was trained and validated on separate data sets of confocal and split detector AOSLO images with results showing performance that closely mimics the gold standard manual process. Further, without any need for algorithmic modifications for a specific AOSLO imaging system, our fully-automated multi-modality CNN-based cone detection method resulted in comparable results to previous automatic cone segmentation methods which utilized ad hoc rules for different applications. We have made free open-source software for the proposed method and the corresponding training and testing datasets available online.

## Introduction

The structure of the retinal photoreceptor mosaic has long been of scientific and clinical interest^1^. A key group of technologies used for visualizing photoreceptors *in vivo* is adaptive optics (AO) ophthalmoscopy^2–9^. AO retinal images have been used to analyze various facets of normal^2, 10–16^ and pathologic photoreceptor mosaics^17–24^. The most widely used of these technologies is the confocal AO scanning light ophthalmoscope (AOSLO)^3^, which is capable of visualizing the smallest photoreceptors, rods and foveal cones^13^ (indeed, AO enhanced optical coherence tomography systems may also visualize rods and foveal cones^25^). More recently, a new generation of AOSLO modalities has been developed that takes advantage of non-confocal information that is lost in confocal systems^26–29^. Non-confocal split detector AOSLO has been shown to provide complementary information to that obtained from confocal AOSLO. It has been suggested that combining multiple modalities could be beneficial for accurately analyzing photoreceptor mosaic properties^30^, or for improving the performance of image processing applications such as registration^31^.

**Figure 1:**
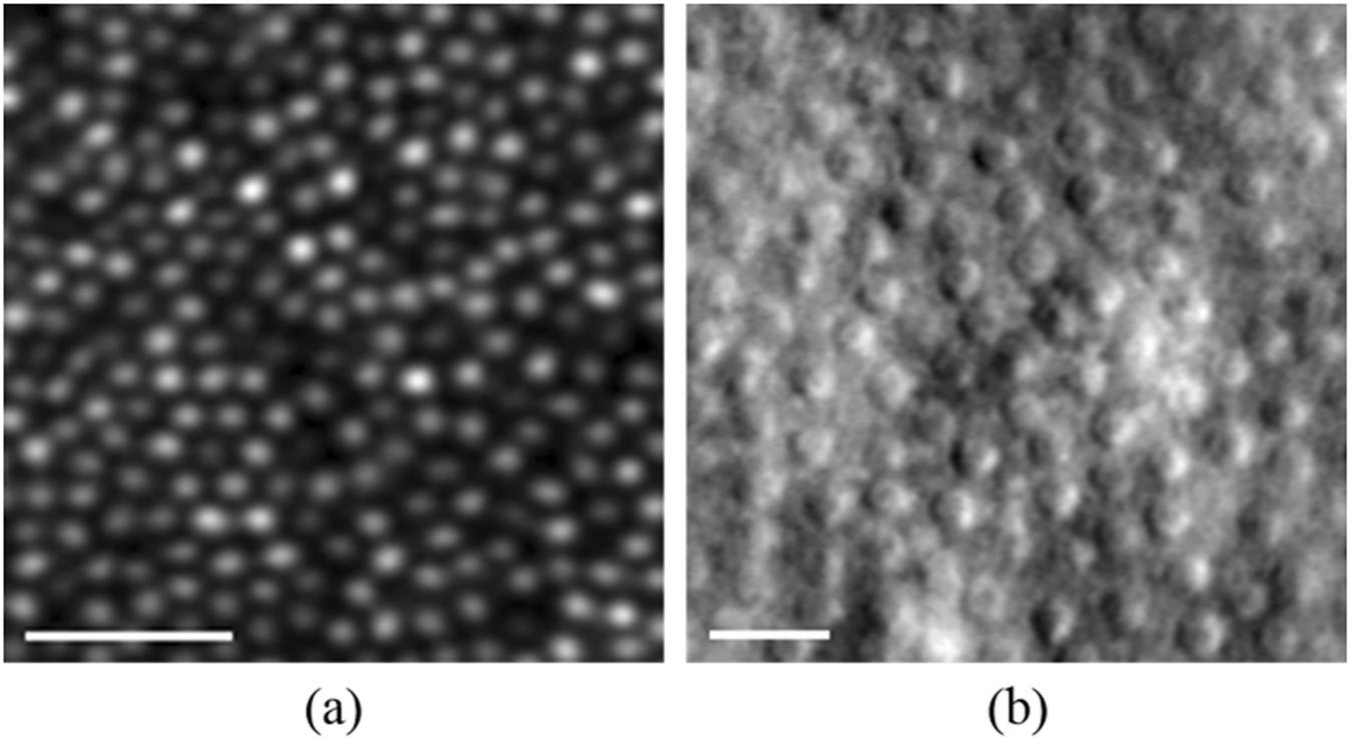
AOSLO cone imaging. (**a**) Confocal AOSLO image at 0.65° from the fovea. (**b**) Split detector AOSLO image at approximately 8° from the fovea. *Scale bars*: 20 μm.

To quantify geometrical properties of the photoreceptor mosaic, often the locations of each individual cone in an image must be found. Since manual grading of these images is subjective and time consuming, several automated methods for detecting cones in AO images have been developed^32^. These automated algorithms use techniques including local intensity maxima detection^33–36^, model based correlation^37^, circular Hough transform^38^, graph-theory and dynamic programming (GTDP)^39^, and estimation of cone spatial frequency^40–42^. These fixed mathematical model based methods have shown good performance for the problems they were designed for (e.g. specific imaging modality, resolution, cone density, etc.). However, reliance on ad hoc rules and specific algorithmic parameters does not allow them to become generalizable to alternative imaging conditions. As an example, in our previous publications, despite developing a highly accurate segmentation method for confocal AOSLO images, we had to devise a completely new segmentation method in order to detect cones in split detector AOSLO images due to the disparity in cone appearances between the two modalities, as can be seen in Fig. 1.

Novel machine learning techniques provide an alternative approach where features of interest are learned directly from data, allowing for a higher degree of adaptability^43^. Unlike classic model-based techniques, one can conceptually use the same core machine learning algorithm for a variety of image segmentation problems by using different training images. Of note, deep convolution neural networks (CNNs)^44–47^ have shown high performance in a multitude of image analysis tasks including over a range of fields, including ophthalmic imaging. CNNs have been used for the segmentation of retinal blood vessels^48–50^, detection of diabetic retinopathy^51^ and hemorrhage^52^ in retinal fundus images, and classification of pathology^53^ or segmentation of retinal layers^54^ in optical coherence tomography images.

In this work, we present a fully automatic method for training a CNN from manually segmented AOSLO images, and then for using the trained CNN in order to detect cones in previously unseen images. We validated the performance of our method on two separate data sets from confocal and split detector AOSLO systems. To promote future research, we provide an open-source MATLAB implementation of our algorithm.

## Methods

**Figure 2:**
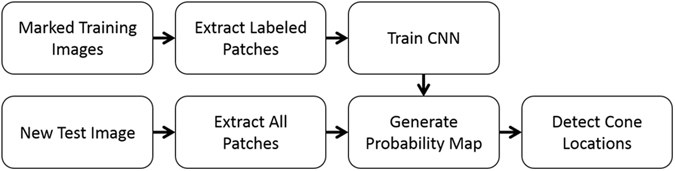
CNN based cone detection algorithm schematic.

Our CNN based cone detection method is outlined in Fig. 2. In brief, we first take a data set of images from an imaging modality (e.g. confocal AOSLO) with manually marked cones and extract patches centered around cone and non-cone locations using the markings. We then use these patches to train a CNN classifier. To detect cones in previously unseen images from this imaging modality, we then classify overlapping patches centered at each pixel to create a probability map. This probability map is then processed to locate the individual cone locations. To detect cones in an alternative AOSLO modality (e.g. split detector AOSLO), all that needs to be done is to exchange the previous training dataset with one from the new imaging modality.

### Data Sets

We used two separate data sets for the confocal and split detector AOSLO cases. These images were previously used for development and validation of cone segmentation algorithms^39, 42^.

The confocal AOSLO experiments were based on the Garrioch *et al*.^36^ data set, which was later used by Chiu *et al*.^39^ to train and validate the GTDP cone segmentation method. In brief, this data set consists of 840 images (150 × 150 pixels) from 21 subjects (20 normal and one subject with deuteranopia) acquired with a previously described confocal AOSLO system^8, 13^ with a 0.96° field-of-view. For each subject, four positions 0.65° from the center of fixation (bottom left, bottom right, top left, and top right) were imaged, and 10 averaged images were acquired per location. Axial length measurements were acquired with an IOL Master (Carl Zeiss Meditec, Dublin, CA) and used to determine the lateral resolution of the images. A region-of-interest (ROI) of size 150 × 150 pixels was extracted from the center of each image and used for analysis. The ROIs’ size ranged from 58 × 58 μm^2^ to 71 × 71 μm^2^. An expert grader semi-automatically marked cone photoreceptors in all images^39^.

The split detector AOSLO experiments used an extension of the data set used by Cunefare *et al*. for training and validating the AFLD cone detection method^42^. The original set contained 112 images (average image size of 208 × 208 pixels) from 14 subjects with normal vision obtained from the Advanced Ocular Imaging Program image bank (MCW, Milwaukee, Wisconsin). These images were acquired using a previously described split detector AOSLO system^8, 28^ with a 1.0° field-of-view. For each subject, eight images were acquired from a single randomly selected meridian (superior, temporal, inferior, and nasal) over a range of eccentricities (500 to 2800 µm). Lateral scale/sampling for each subject was determined using an IOL Master (Carl Zeiss Meditec Inc., Dublin, California, USA). An ROI was extracted from each image and used for analysis. The size of each ROI was chosen as a function of retinal eccentricity so that the ROI would contain approximately 100 cones^55^. In addition to the published data set in Cunefare *et al*.^42^, we acquired a new set of 152 split detector images (ROIs with an average size of 216 × 216 pixels) from four subjects with normal vision using the same split detector system. The ROIs’ size across the entire split detector set ranged from 93 × 93 μm^2^ to 111 × 111 μm^2^. An expert grader manually marked cone photoreceptors in all images.

We learned separate CNN weights and algorithmic parameters for the cases of confocal images, split detector images, and a combination of both modalities. For the case of confocal AOSLO imaging, we used 200 images from 5 subjects for training the CNN, and 640 images from the remaining 16 subjects for validating the proposed method. The images from the subject with deuteranopia were used in the validation data set. For the case of split detector AOSLO, we used 184 images from 8 subjects for training. We evaluated the performance of the CNN by using the same 80 images from 10 subjects that were used for validation of our previous study^42^. For the combined case, we added both the confocal and split detector training data sets to form a single mixed training data set. In this case, the network was blinded to the type of AOSLO image. There was no overlap in subjects between the training and validation sets for either the confocal or split detector cases.

### Image Preprocessing and Patch extraction

**Figure 3:**
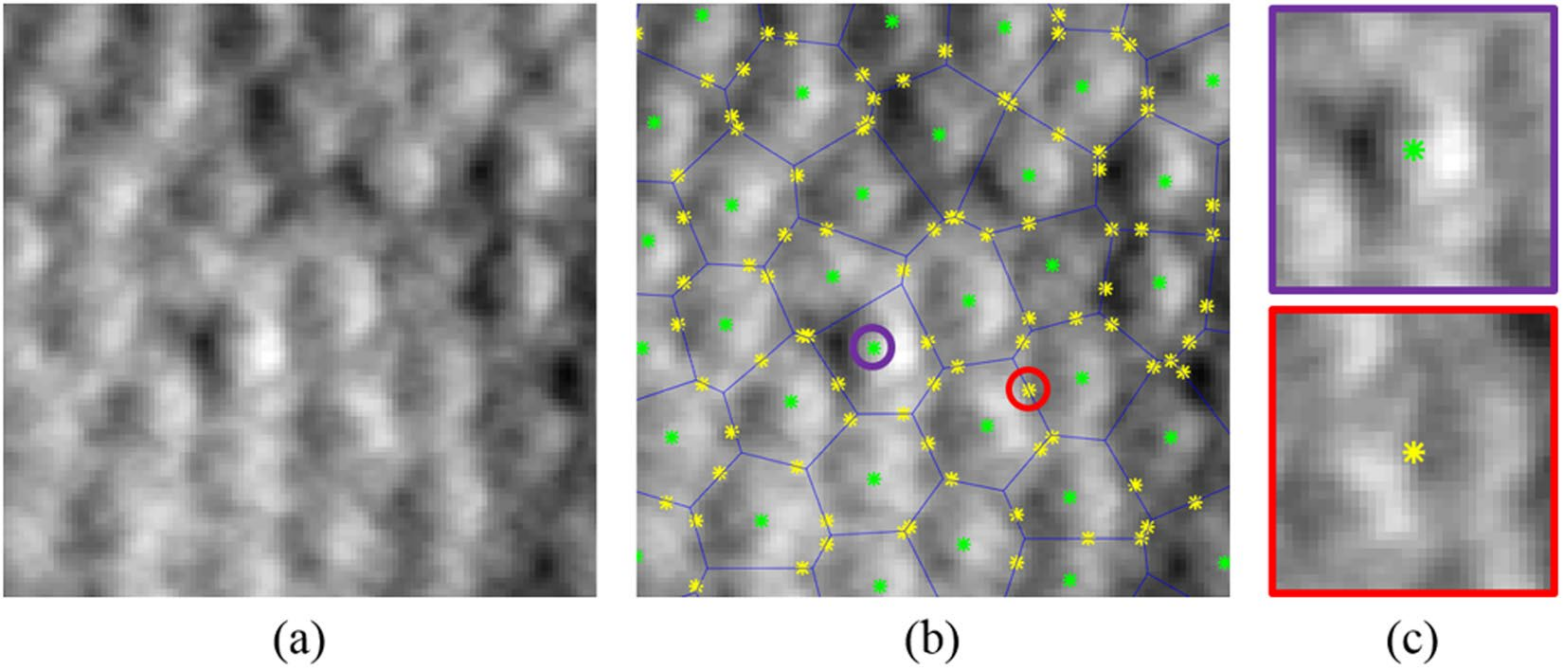
Extraction of labeled patches from cone images. (**a**) Original cropped split detector AOSLO image. (**b**) Image (**a**) with Voronoi diagram overlain in blue. Manually marked cones are shown in green and randomly generated locations along Voronoi edges in yellow. (**c**) Example cone (top-purple) and non-cone (bottom-red) patches (size 33 × 33 pixels) from positions circled in (**b**) with center marked.

We normalized all images so that their intensity values stretched between 0 and 255. Each neural network was trained by first extracting patches centered on cone and non-cone pixel locations. For the set corresponding to cone locations, we extracted patches of size 33 × 33 pixels centered on every manually marked position in the training images. The patch size was chosen empirically to be large enough to contain cones and their surrounding features. Our training data sets did not include direct manual markings for the non-cone areas. Thus, we devised a simple technique using Voronoi diagrams^33^ to select the non-cone locations. First, the manually marked cone positions are used to generate a Voronoi diagram, as shown in Fig. 3(b), where each cell consists of all positions that have a smaller Euclidian distance to the contained cone position than any other cone position. The boundaries between these cells are called Voronoi edges, which we assumed to correspond with the space between individual cones. Subsequently, in order to generate the non-cone set, we randomly selected a single point from each Voronoi edge for all training images, rounded to the nearest pixel value, and then extracted a 33 × 33 pixel patch around this location (Fig. 3(c)). Patches that would extended outside of the image were excluded. Over 34000 cone and 88000 non-cone patches were extracted from the confocal training data set, and over 20000 cone and 49000 non-cone patches were extracted from the split detector training set.

### Convolutional Neural Network

**Figure 4:**
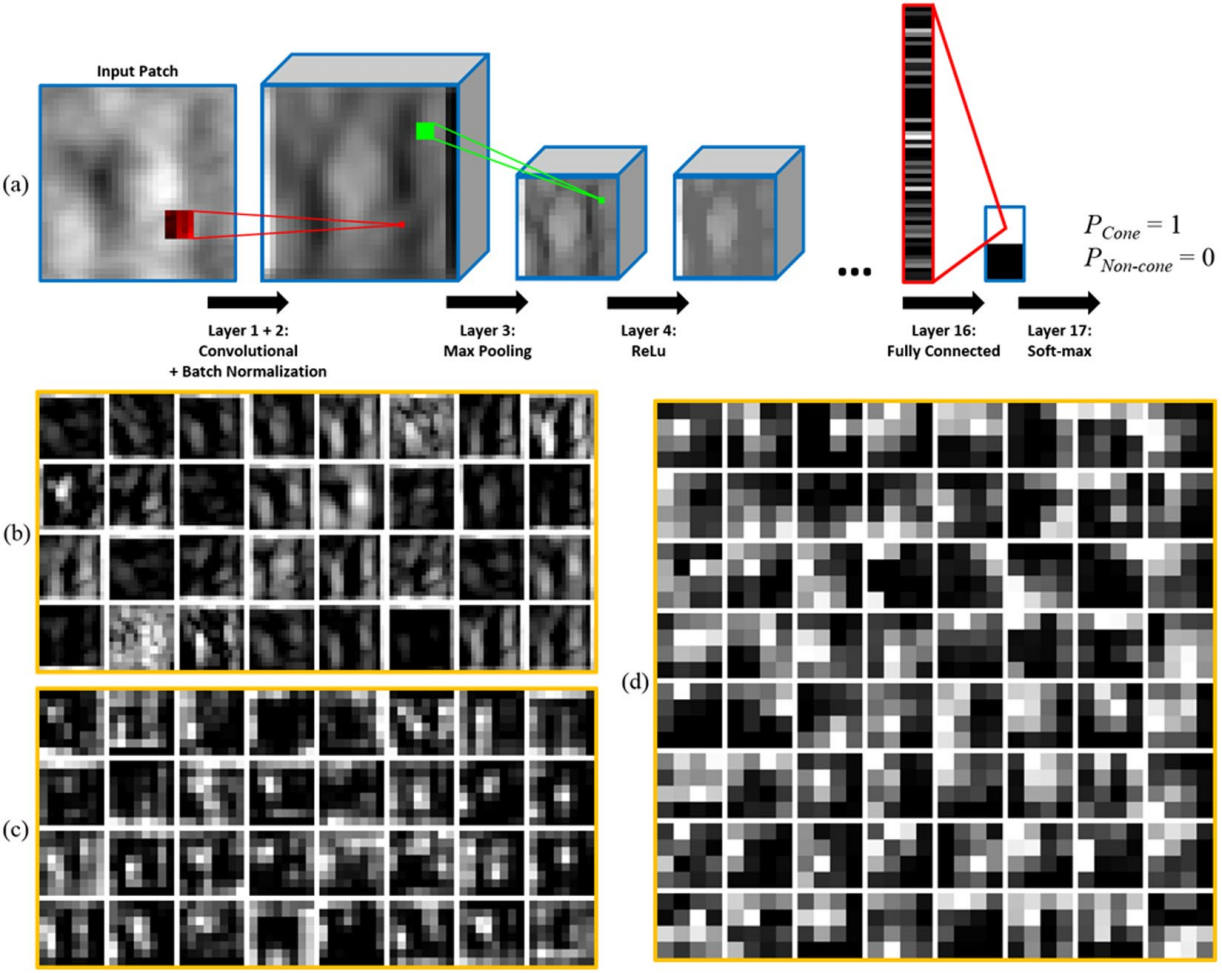
Convolutional network properties. (**a**) Visualization of convolutional, pooling, ReLu, fully connected, and soft-max layers. (**b**–**d**) Output feature maps from (**b**) Layer 4 (**c**) Layer 8, and (**d**) Layer 12 when the input patch from (**a**) is processed by the trained SD-CNN.

We slightly modified a Cifar^44^ CNN architecture implemented in the MatConvNet^56^ CNN toolbox. In general, a simple CNN accepts an input image or patch and applies a sequence of transforming layers in order to extract features and finally classify the input. Table 1 shows the sequence of layers used in our network. Convolutional layers convolve an input of size *W* × *H* × *D* (before padding) with *N* kernels of size *w* × *h* × *D* to get an output of size *W* × *H* × *N*. The output can be thought of as a stack of *N* feature maps, each generated by the corresponding kernel. For each of these feature maps, the CNN adds a potentially different bias value. The pooling layers apply either a max or average operation over the first two dimensions in a 3 × *3* window while down sampling the input by a factor of 2. This lowers the computational burden and improves robustness to small image distortions by increasing spatial invariance^57^. Rectified linear units (ReLU) layers^58^ transform their inputs by setting all negative values to 0. ReLU layers are used to speed up the training process^44^ and improve the performance of the network by adding non-linearity^57^. Batch normalization layers^59^ normalize their inputs based on mean and variance statistics. These layers are used to reduce internal covariate shift which speeds up the training process and helps prevent overfitting. Fully connected layers consolidate all information from their preceding layer. Each output node in a fully connected layer is obtained by a weighted combination of all values from the previous layer with an additional bias term added for each node. The final fully connected layer provides a score for each class (cone and non-cone), which are input into a soft-max layer^60^ that outputs the probability of the original patch belonging to each class. Figure 4(a) provides an illustrative example of all layer types used in our CNN.

**Table 1:** Architecture of the CNN.

Layer number	Type	Input size	Filter size	Stride	Number of kernels/nodes
**1**	Convolution	33 × 33 × 1	5 × 5 × 1	1	32
**2**	Batch Normalization	33 × 33 × 32	—	—	—
**3**	Max Pooling	33 × 33 × 32	3 × 3	2	—
**4**	ReLU	16 × 16 × 32	—	—	—
**5**	Convolution	16 × 16 × 32	5 × 5 × 32	1	32
**6**	Batch Normalization	16 × 16 × 32	—	—	—
**7**	ReLU	16 × 16 × 32	—	—	—
**8**	Average Pooling	16 × 16 × 32	3 × 3	2	—
**9**	Convolution	8 × 8 × 32	5 × 5 × 32	1	64
**10**	Batch Normalization	8 × 8 × 64	—	—	—
**11**	ReLU	8 × 8 × 64	—	—	—
**12**	Average Pooling	8 × 8 × 64	3 × 3	2	—
**13**	Fully Connected	4 × 4 × 64	4 × 4 × 64	—	64
**14**	Batch Normalization	1 × 1 × 64	—	—	—
**15**	ReLU	1 × 1 × 64	—	—	—
**16**	Fully Connected	1 × 1 × 64	1 × 1 × 64	—	2
**17**	Soft-max	1 × 1 × 2	—	—	—

In order for the CNNs to be effective, the convolutional and fully connected weights and biases must be learned. We trained separate networks for the confocal and split detector cases, which we label the confocal CNN (C-CNN) and split detector CNN (SD-CNN). Each network was trained using the extracted training patches and their class labels as inputs. Additionally, we trained a combined network, which we labeled the mixed CNN (M-CNN), using the patches from both the confocal and split detector training images without providing the network any indication which modality the patches originated from. The initial weight parameters are randomly initialized, and the initial biases are set to zero. Then, the weights and biases are refined through stochastic gradient descent and back-propagation^46^. The training data is split into mini-batches with 100 patches per mini-batch, and each iteration of the gradient descent occurs over one mini-batch. We repeated this process for all batches spanning the training data set (called an epoch^48^), and trained over 45 epochs for both networks. The weight learning rate was initially set to 0. 001 for all layers except the last fully connected layer (layer 12) where it was set to 0.0001. Bias learning rates were similarly set to 0.1 for all layers except the last fully connected layer where it was set to 0.01. All learning rates were decreased by a factor of 10 twice over training, at epochs 31 and 41. Weight decay was left at the default value of 0.0001 in the MatConvNet^56^ CNN toolbox. Figure 4(b–d) displays the feature maps output after each sequence of convolutional, batch normalization, pooling, and ReLU layers in the trained SD-CNN for a single cone input patch.

### Cone Detection

**Figure 5:**
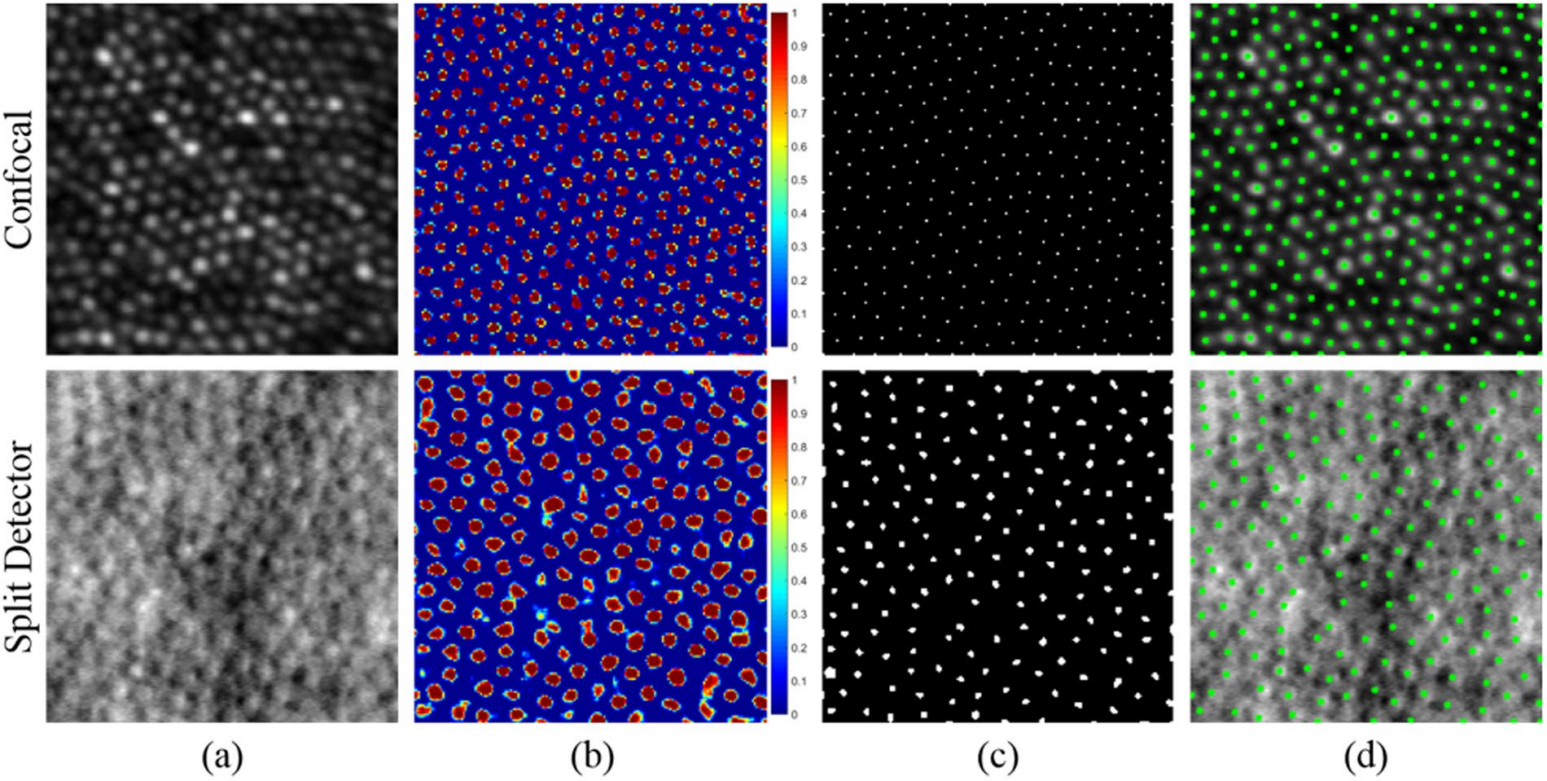
Detection of cones in confocal (top row) and split detector (bottom row) AOSLO images. (**a**) Original images. (**b**) Probability maps generated from (**a**) using the C-CNN (top) and SD-CNN (bottom). (**c**) Extended maxima of (**b**). (**d**) Detected cones marked in green on the image shown in (**a**).

After training the CNNs, we used them to find cone locations within an image. To do this, we first extracted a 33 × 33 pixel patch around every pixel in the image after normalizing the image’s intensity values. Symmetric padding by mirroring the pixel values at the edge by half of the patch length (16 pixels) was used for patches that extended beyond the edges of the image. We then used the corresponding CNN to determine the probability that each patch is centered on a cone. The associated probability for each patch is then used to construct a probability map with the same dimensions as the original image, as shown in Fig. 5(b). We smoothed the map with a Gaussian filter with standard deviation *σ*. We then applied the extended-maxima transform using MATLAB’s *imextendedmax* function^61^, which finds maximal regions where the height difference in the region is less than or equal to *H*. This results in a binary image as shown in Fig. 5(c). We then found all connected clusters in the binary image to use as candidates for cone locations, and eliminated weak candidates by removing any cluster whose maximum value in the filtered probability map is less than a threshold *T*. Finally, the locations of cones in the image are determined by finding the centers of the remaining clusters (Fig. 5(d)). The values *σ*, *H*, and *T* were automatically chosen to be 1.3, 0, and 0.3 for the C-CNN case; 2, 0.1, and 0.5 for the SD-CNN case; and 0.4, 0.25, and 0.9 for the M-CNN case. These automatically chosen parameters were learned by maximizing the average Dice’s coefficient (as explained in the following section) across the training images over a set of potential parameter combinations.

### Validation

We validated the results of our method by comparing to the current gold standard of manual grading across the validation data sets. In the case of M-CNN, we provided no explicit information to the network about the type of the AOSLO image to be tested (confocal or split-detector). Additionally, we compared the performance of our CNN based method to the performance of the GTDP cone detection method^39^ for the confocal set, and to the AFLD method^42^ for the split detector set. To quantify the performance of the automatic methods, we matched automatically marked cones to manually marked cones one-to-one. In brief, an automatic cone was considered a true positive if it was located within some distance *d* of a manually marked cone. The value *d* was set to 0.75 of the median spacing between manually marked cones in the image. Automatically detected cones that were not matched to a manually marked cone were considered as false positives, and manually marked cones that did not have a matching automatically detected cone were considered as false negatives. If a manually marked cone was matched to more than one automatically marked cone, only the one with the smallest distance was considered a true positive, and the remaining were considered false positives. Finally, we removed manually and automatically marked cones that were within 7 pixels of the edges of the image to remove border artifacts. For each image, the number of automatically marked cones (N_Automatic_) and manually marked cones (N_Manual_) can then be broken down into the number of true positives (N_TP_), false positives (N_FP_), and false negatives (N_FN_) as:

1 $${{\rm{N}}}_{{\rm{Automatic}}}={{\rm{N}}}_{{\rm{TP}}}+{{\rm{N}}}_{{\rm{FP}}},$$

2 $${{\rm{N}}}_{{\rm{Manual}}}={{\rm{N}}}_{{\rm{TP}}}+{{\rm{N}}}_{{\rm{FN}}}.$$

In order to evaluate the performance of the automatic methods, we then calculate the true positive rate, false discovery rate, and Dice’s coefficient^62, 63^ for each image as:

3 $${\rm{T}}{\rm{r}}{\rm{u}}{\rm{e}}\,{\rm{p}}{\rm{o}}{\rm{s}}{\rm{i}}{\rm{t}}{\rm{i}}{\rm{v}}{\rm{e}}\,{\rm{r}}{\rm{a}}{\rm{t}}{\rm{e}}={{\rm{N}}}_{{\rm{T}}{\rm{P}}}/{{\rm{N}}}_{{\rm{M}}{\rm{a}}{\rm{n}}{\rm{u}}{\rm{a}}{\rm{l}}},$$

4 $${\rm{F}}{\rm{a}}{\rm{l}}{\rm{s}}{\rm{e}}\,{\rm{d}}{\rm{i}}{\rm{s}}{\rm{c}}{\rm{o}}{\rm{v}}{\rm{e}}{\rm{r}}{\rm{y}}\,{\rm{r}}{\rm{a}}{\rm{t}}{\rm{e}}={{\rm{N}}}_{{\rm{F}}{\rm{P}}}/{{\rm{N}}}_{{\rm{A}}{\rm{u}}{\rm{t}}{\rm{o}}{\rm{m}}{\rm{a}}{\rm{t}}{\rm{i}}{\rm{c}}},$$

5 $$\mathrm{Dice}\mbox{'}{\rm{s}}\,{\rm{coefficient}}={{\rm{2N}}}_{{\rm{TP}}}/{(N}_{{\rm{Manual}}}+{{\rm{N}}}_{{\rm{Automatic}}}){\rm{.}}$$

Additionally, we also evaluated cone density measurements from each method, which is a commonly used quantitative metric for analyzing photoreceptor mosaics^64^. Cone density is defined as the ratio of the number of cones in an image to the area of that image. We compared cone densities calculated from each automatic method to the cone density from manual grading using Bland-Altman analysis^65^.

### Data Availability

The datasets generated during and analyzed during the current study are available at https://github.com/DavidCunefare/CNN-Cone-Detection. These include both training and validation datasets for confocal and split detector AOSLO and the corresponding open source software.

## Results

We implemented the CNN based detection method in MATLAB 2016b (The MathWorks, Natick, MA) with MatConvNet^56^ 1.0-beta23. We ran the algorithm on a desktop PC with an i7-5930K CPU at 3.5 GHz, 64 GB of RAM, and a GeForce GTX TITAN X GPU. The average run time for our CNN based detection method on a new image after training was 6 seconds per image for the confocal AOSLO data set (image size of 150 by 150 pixels) and 12 seconds per image for the split detector AOSLO data set (average image size of 206.5 by 206.5 pixels). These times were the same regardless of whether the C-CNN, SD-CNN, or M-CNN was used. The total training time was under 3 hours for the C-CNN and SD-CNN, and under 6 hours for the M-CNN. This included the time for extracting training patches, training the CNN, saving the probability maps of all training images, and choosing the detection parameters. Note that this offline training only needs to be completed once.

**Figure 6:**
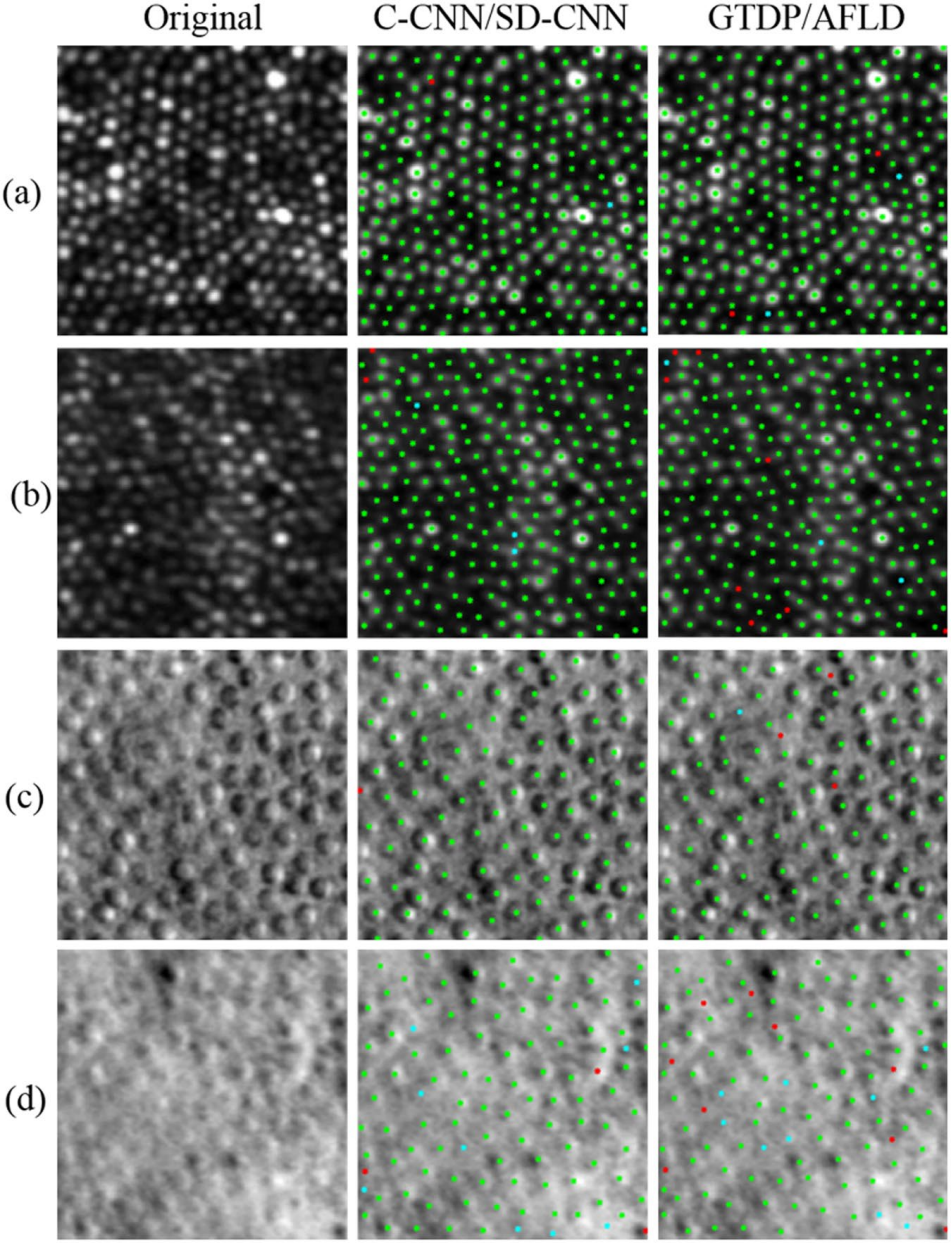
Performance of the automated algorithms on confocal (**a**,**b**) and split detector (**c**,**d**) images. Original Images are shown in the left column, C-CNN for confocal and SD-CNN for split detector cone detection results in the middle column, and AFLD for split detector and GTDP for confocal results in the right column. Green points denote true positives, cyan denotes false negatives, and red denotes false positives. Dice’s coefficients for our CNN based method are 0.994 in (**a**), 0.990 in (**b**), 0.995 in (**c**), and 0.941 in (**d**). Dice’s coefficient for the GTDP method are 0.992 in (**a**) and 0.978 in (**b**), and for the AFLD method are 0.979 in (**c**) and 0.911 in (**d**).

Figure 6 displays the results of the automated algorithms in comparison to manual grading in four examples. In the marked images, a green point indicates an automatically detected cone that was matched to a manually marked cone (true positive), a cyan point indicates a cone missed by the automatic algorithm (false negative), and a red point indicates an automatic marking with no corresponding manually marked cone (false positive).

The performances of the automated algorithms in comparison to manual grading are summarized in Tables 2 and 3. Table 2 reports the performance of the C-CNN, M-CNN and GTDP methods across the confocal AOSLO validation data set, and Table 3 reports the performance of the SD-CNN, M-CNN and AFLD methods across the split detector AOSLO validation data set. The median Dice’s coefficients across the confocal validation set were 0.993 for the C-CNN, 0.990 for the M-CNN, and 0.991 for the GTDP methods. The median Dice’s coefficients across the split detector validation set were 0.970 for the SD-CNN, 0.968 for the M-CNN, and 0.958 for the AFLD methods. As noted previously, one subject used in the confocal validation data set had deuteranopia. The average true positive rate, false discovery rate, and Dice’s coefficient for this subject using the C-CNN were found to be 0.993, 0.007, and 0.993, respectively. It can be seen that the performance of the general purpose CNN method is very comparable to the custom designed GTDP and AFLD techniques.

**Table 2:** Average performance of the C-CNN, M-CNN, and GTDP methods with respect to manual marking across the confocal validation data set (standard deviations shown in parenthesis).

	True positive rate	False discovery rate	Dice’s coefficient
C-CNN	0.989 (0.012)	0.008 (0.014)	0.990 (0.010)
M-CNN	0.975 (0.026)	0.003 (0.007)	0.986 (0.015)
GTDP	0.990 (0.011)	0.015 (0.018)	0.988 (0.012)

**Table 3:** Average performance of the SD-CNN, M-CNN, and AFLD methods with respect to manual marking across the split detector validation data set (standard deviations shown in parenthesis).

	True positive rate	False discovery rate	Dice’s coefficient
SD-CNN	0.943 (0.075)	0.027 (0.034)	0.955 (0.045)
M-CNN	0.949 (0.057)	0.034 (0.036)	0.956 (0.034)
AFLD	0.961 (0.031)	0.054 (0.045)	0.952 (0.028)

**Figure 7:**
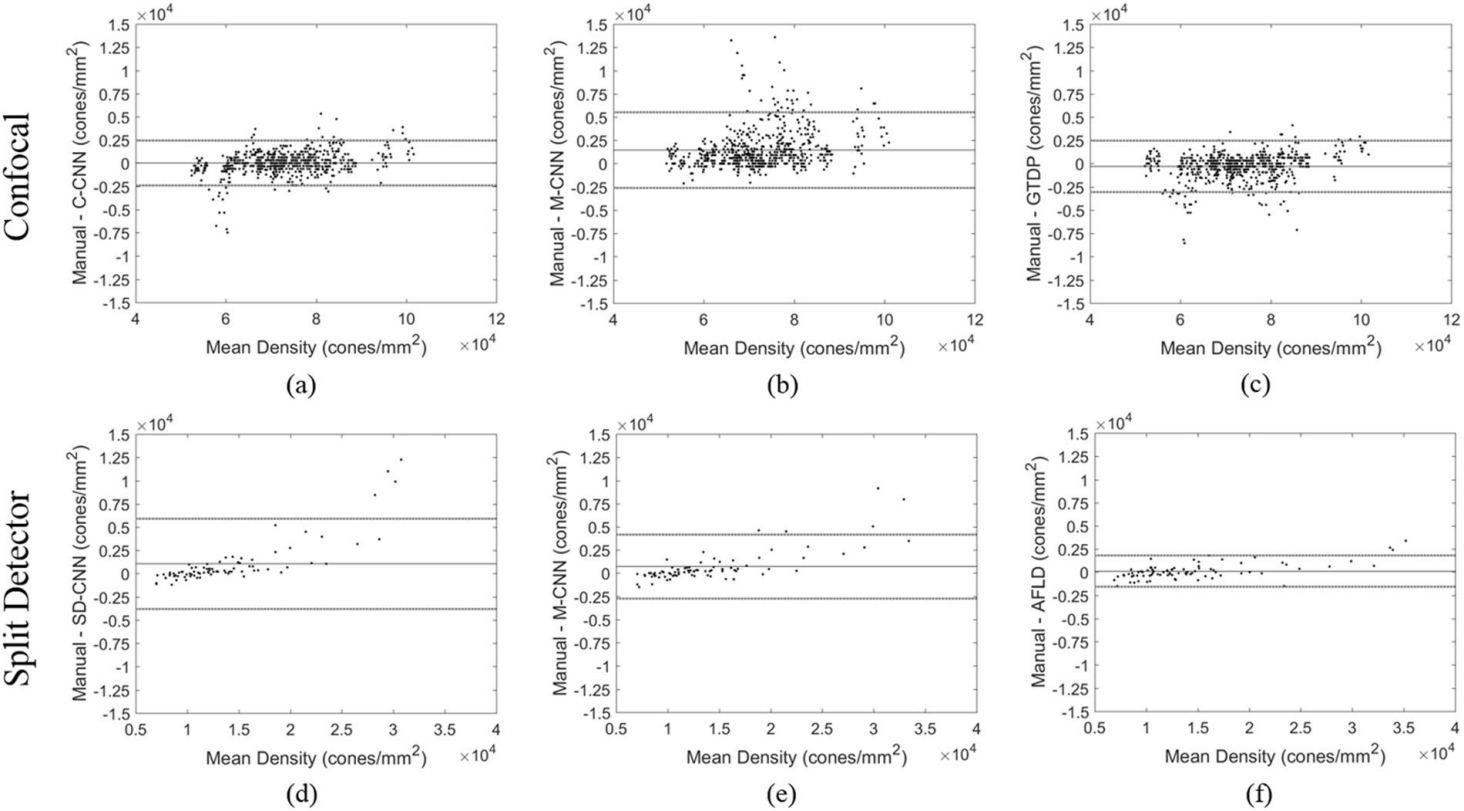
Bland-Altman plots comparing cone density for (**a**) manual – C-CNN on confocal, (**b**) manual – M-CNN on confocal, (**c**) manual – GTDP on confocal, (**d**) manual – SD-CNN on split detector, (**e**) manual – M-CNN on split detector, and (**f**) manual – AFLD on split detector. The solid black line shows the expected value of the difference, and the dotted lines show the 95% confidence limits of agreement.

Bland-Altman plots comparing cone density measurements between the automatic methods and manual grading are shown in Fig. 7. The central solid line shows the average difference between methods, and the surrounding dotted lines show the 95% confidence limits.

## Discussion

In this work, we developed an automatic CNN based method for detecting cone photoreceptors in AOSLO images. Using manually marked images from a particular AOSLO imaging modality, our method can train a CNN to extract features of interest and classify cones in unseen images from the same imaging modality. We tested our method on images from confocal and split detector AOSLO systems, and showed that or method had good agreement with the current gold standard of manual marking for both modalities. We have made the open-source code for our CNN based method freely available online to allow other researchers to test and modify the algorithm for their specific applications.

As can be seen in Tables 2 and 3, our CNN based algorithm performed comparably to the state-of-the-art GTDP and AFLD cone detection methods with respect to manual grading. This is highly encouraging, because the noted previous techniques heavily utilized modality specific ad hoc rules, which limited their application to other imaging scenarios and require further algorithm modification and development for new imaging scenarios. Our CNN approach, on the other hand, required no ad hoc rules, and was applied without any algorithmic modification to confocal and split detector AOSLO images. All that was needed to adapt the algorithm to confocal and split detector AOSLO was the corresponding training datasets. Additionally, in the case of the M-CNN, our algorithm was able to learn network filter weights to accurately detect cones in split detector and confocal images without any knowledge of which modality the image was acquired with.

For both the confocal and split detector AOSLO data sets, our CNN-based method had a slightly worse true positive rate but a slightly better false discovery rate than the other corresponding algorithm. This is also reflected in Fig. 7, where the CNN based method is biased towards underestimating the number of cones, especially in images with higher cone densities. This could be improved by using training data sets with a larger representation of images with high cone densities. Moreover, poor inter-observer agreement in grading AOSLO images^66^ can negatively affect the performance of learning based methods such as CNN. Utilization of datasets graded by multiple observers is expected to improve performance.

The quantitative performance metrics presented for the GTDP and AFLD algorithms have negligible differences in comparison to those reported in Chiu *et al*.^39^ and Cunefare *et al*.^42^, respectively, for two reasons. First, these differences arise from changes made to the methods for quantifying performance (e.g. the number of cropped pixels in the boundaries) so that a uniform method could be used for both data sets. Second, we used a subset of the validation set used in Chiu *et al*.^39^ for training the CNN, which was naturally excluded from the validation data set in this work.

It should be noted that there is space for improvement our proposed algorithm. We choose the network architecture and hyper-parameters empirically to provide good performance for both confocal and split detector AOSLO images. It is possible these parameters could be optimized to provide better performance, especially for a single imaging modality. Additionally, we expect to further improve the performance of the method for specific applications by applying custom pre-processing steps such as adaptive denoising^67^.

Since our CNN based method learned to detect cones directly from training data, it can be easily extended to other imaging modalities or imaging conditions with different features (e.g. images from different retinal eccentricities that may contain rods or images of diseased eyes). Extending the algorithm to images from subjects with retinal pathologies is of particular importance. Several retinal diseases lead to alterations of the cone mosaic (e.g. age-related macular degeneration, achromatopsia, retinitis pigmentosa, and Usher syndrome), and quantitative analysis of these mosaics captured with AOSLO is potentially useful for the characterization, early diagnosis, and prognosis of these diseases^30, 68^. By sharing the open source software of this paper freely available online, we encourage our colleagues to test this algorithm for new imaging conditions. Of course, due to the variability of cone photoreceptor manifestation in AOSLO images of different diseases or eccentricities, we expect that utilizing appropriate new training data sets that match the test data set (e.g. training and testing on the same eccentricity or disease condition) will improve the performance of the algorithm. Detailed evaluation of our algorithm’s performance for different diseases, imaging eccentricities, and images containing rod photoreceptors is part of our ongoing work.
